# Characterization of P2X7R and Its Function in the Macrophages of ayu, *Plecoglossus altivelis*

**DOI:** 10.1371/journal.pone.0057505

**Published:** 2013-02-21

**Authors:** Yu-Qing He, Jiong Chen, Xin-Jiang Lu, Yu-Hong Shi

**Affiliations:** School of Marine Sciences, Ningbo University, Ningbo, China; Chang Gung University, Taiwan

## Abstract

P2X purinoceptor 7 (P2X7R), an ATP-gated ion channel, plays an important role during the innate immune response in mammals. However, relatively little is known about the role of P2X7R in the fish immune system. Here, we cloned a cDNA sequence encoding ayu (*Plecoglossus altivelis*) P2X7R (aP2X7R). The predicted protein was composed of 574 amino acid residues with a P2X family signature, two transmembrane domains, and a long C-terminal. aP2X7R transcripts were mainly distributed in ayu immune tissues and significantly increased in all tested tissues and in macrophages after *Listonella anguillarum* infection. The aP2X7R protein was upregulated significantly in macrophages upon bacterial challenge. An antibody against the ectodomain of aP2X7R (aEPAb) and an antagonist (oATP) were used to block aP2X7R. aP2X7R siRNA was also used to knockdown the receptor expression in ayu macrophages. Cell death induced by ATP was significantly inhibited in ayu macrophages after aEPAb, oATP, or siRNA treatment. Moreover, aP2X7R ablation also resulted in suppression of phagocytic activity and ATP-induced bacterial killing in ayu macrophages. Our results indicated that aP2X7R was upregulated after infection and mediated cell death, phagocytosis, and bacterial killing of ayu macrophages.

## Introduction

The P2X purinoceptor 7 (P2X7R), an ionotropic receptor gated by adenosine triphosphate (ATP), was first identified in rat [1]. It is widely distributed in nearly all tissues and organs, with the highest expression observed in macrophages [2], [3]. P2X7R possesses two transmembrane domains, intracellular N- and C-termini, and a long carboxyl terminus, containing five ATP ligand-binding motifs in the ectodomain and one conserved LPS-binding motif in the C-terminus [4]. Activation of P2X7R leads to a variety of downstream events, including Ca^2+^ influx [5], nonselective large pore formation [1], cell death [6], interleukin (IL)-1β release [6], membrane permeabilization [5], and reactive oxygen species release [7].

P2X7R expression has been reported to be upregulated upon pathogen infection [8]. Moreover, P2X7R is involved in the functional regulation of immune cells, and activation of P2X7R strongly enhances intracellular bacterial killing in macrophages and induces macrophage death [9]. Transfection with P2X7R confers phagocytic abilities on nonphagocytic HEK-293 cells, while blocking P2X7R expression by siRNA significantly reduces the phagocytic abilities of THP-1 cells, a monocytic leukemia cell line [10]. Furthermore, ATP can activate P2X7R to release IL-1β in human monocytes priming by lipopolysaccharide [11]. Thus, P2X7R plays important roles in innate immunity in mammals.

Because of the economic and environmental impact of fish and diseases in fish, many studies conducted over the past decade have studied the immune system of fish [12]. However, little information is available on the functions and characteristics of fish P2X7R, although the function of P2X7R is known to be important in mammalian macrophages [7]–[9]. Therefore, it is necessary to investigate the function of P2X7R in the fish immune system. Until recently, only zebrafish (*Danio rerio*) [13] and gilthead seabream (*Sparus aurata*) [14] P2X7R genes had been cloned in fish. P2X7R does not mediate IL-1β release in the gilthead seabream, which is different from that reported in mammals [14]. However, the role of P2X7R in regulating the fish immune system still remains obscure.

The ayu (*Plecoglossus altivelis*) is an economically important fish species in Asia. Intensive ayu farming has promoted the growth of many bacterial and viral diseases that have resulted in both production and animal welfare problems [15], [16]. Hence, because of the economic importance of this fish, it seems especially important to study the immune response of fish against microbiological pathogens. In this work, we aimed to clone ayu P2X7R (aP2X7R) cDNA, study the expression and functional responses of aP2X7R during *Listonella anguillarum* challenge, and its potential role in macrophages.

## Materials and Methods

### Fish rearing

About 120 healthy ayu, weighing 40–50 g each, were purchased from a fishery in Fuxi, Ninghai County, Ningbo City, China. These fish were maintained and acclimatized in aerated fresh water at 20–22°C with regular feeding as previously described [17]. Only healthy fish, without any pathological signs, were used in the study. All animal work in this paper was conducted according to relevant national and international guidelines. All animal care and experimental procedures were approved by the Committee on Animal Care and Use and the Committee on the Ethics of Animal Experiments of Ningbo University.

### Bacterial challenge

*L. anguillarum* challenge in the ayu was performed as described previously [17]. Briefly, overnight cultures of *L. anguillarum* were diluted 1:50 in basic peptone water medium, cultured at 28°C with shaking, and harvested in the logarithmic growth. Cells were washed, resuspended, and adjusted to a final concentration of 1.0×10^6^ colony-forming units (CFU) ml^−1^ in sterile normal saline. 40 fish were intraperitoneally injected with 100 µL of *L. anguillarum* per fish, and 40 other fish were injected with 100 µL of saline per fish as a negative control. Each tank contained 20 bacteria-infected or healthy control fish. Samples of infected and control fish were randomly collected at 0, 4, 8, 12, and 24 h postinjection (hpi), frozen in liquid nitrogen, and stored at −70°C until use.

### Determination of the cDNA sequence of aP2X7R

Total RNAs were extracted from ayu head kidney with RNAiso Reagent (TaKaRa, Dalian, China) following the manufacturer’s instructions and treated with RNase free DNase I. The mRNA in 1 µg total RNA was reverse transcribed using M-MLV reverse transcriptase (TaKaRa) following standard protocols. Based on the partial sequence of aP2X7R, which was obtained from previous transcriptome sequencing, the full-length cDNA sequence was determined using the rapid amplification of cDNA ends (RACE) method [18]. PCR amplification products were sequenced by an ABI 3730 automated sequencer (Invitrogen, Carlsbad, CA, USA).

### Sequence analysis

The similarity of the obtained aP2X7R sequence (accession number: HE984576) with known P2X7R sequences, i.e., human (*Homo sapiens*), Q99572; small-eared galago (*Otolemur garnettii*) XM_003795998; dog (*Canis lupus familiaris*), NM_001113456; horse (*Equus caballus*), XM_001495572; pig (*Sus scrofa*), XM_001926804; cattle (*Bos Taurus*), NM_001206516; rabbit (*Oryctolagus cuniculus*), XM_002719745; mouse (*Mus musculus*) AJ489297; rat (*Rattus norvegicus*), NM_019256; clawed frog (*Xenopus laevis*), AJ345114; chicken (*Gallus gallus*), XM_001235162; green anole (*Anolis carolinensis*), XM_003222779; gilthead seabream (*S. aurata*), AJ887997; and zebrafish (*D. rerio*), AY292647, was analyzed using BLAST (http://blast.ncbi.nlm.nih.gov/Blast.cgi). The cleavage site of signal peptides was predicted by the SignalP 4.0 program (http://www.cbs.dtu.dk/services/SignalP/). The transmembrane helices were predicted by the “DAS”- Transmembrane Prediction server (http://www.sbc.su.se/~miklos/DAS/). Multiple alignments were analyzed using ClustalW (http://clustalw.ddbj.nig.ac.jp/). Phylogenetic and molecular evolutionary analyses were conducted using MEGA version 4 [19].

### Real-time quantitative PCR (RT-qPCR)

Changes in the mRNA expression of aP2X7R following *L. anguillarum* infection were analyzed by RT-qPCR as previously described [17]. RT-qPCR was conducted on an ABI StepOne Real-Time PCR System (Applied Biosystems, USA) using SYBR premix Ex Taq (Perfect Real Time) (TaKaRa) in accordance with the manufacturer’s instructions. To assess PCR efficiency, 10-fold serial dilutions of both aP2X7R and β-actin plasmid cDNA were used to generate a standard curve for each assay plate. According to the standard curve, the PCR efficiency was determined to be 92% and 94% for aP2X7R and β-actin, respectively. After the amplification, melt curves were obtained by slow heating from 60°C to 95°C at 0.1°C/s, with continuous fluorescence collection, confirming that only our specific product peaks were detected. Amplifications with aP-F: 5′-TCCCAGTTCAGACGGACAG-3′ and aP-R: 5′-TTAAGGTGTGGTGTTTGCCA-3′ primers were performed with cDNA from the head kidney, spleen, liver, gill, intestine, heart, and muscle of infected and control fish. As a control, a 231-bp fragment of the housekeeping β-actin gene was amplified from the same cDNA preparations using pActin-F: 5′-TCGTGCGTGACATCAAGGAG-3′ and pActin-R: 5′-CGCACTTCATGATGCTGTTG-3′ primers. The mRNA expression of aP2X7R from macrophages was also detected. Relative gene expression was analyzed by the comparative Ct method (2^-ΔΔCt^ method). The mRNA expression of aP2X7R was normalized against the expression of β-actin. Data were expressed as the mean ± SEM and analyzed by one-way analysis of variance (ANOVA) with SPSS version 13.0 (SPSS Inc., Chicago, IL, USA). Four independent experiments were performed. Differences were considered significant at *P*<0.05.

### Prokaryotic expression of the ectodomain fragment of aP2X7R

Based on the previously determined sequence, a specific primer pair was designed that would amplify a 750-bp fragment comprising amino acids 60–310 of aP2X7R ectodomain peptide (aEP) and included restriction sites for *Nde*I and *Bam*HI (underlined) at the 5′-ends of aEP-F: 5′-CCATATGACAAAGGTCAAGGGAGTGGC-3′ and aEP-R: 5′-CGGATCCTTATCCAAAAGCTTTGTACAG-3′ primers, respectively, to facilitate directional cloning into the pSBET vector. Pfu DNA Polymerase (Fermentas, Vilnius, Lithuania) was used for gene amplification according to the manufacturer’s protocol. BL21 (DE3) *Escherichia coli* transformed with pSBET-aEP plasmid were used for overexpression of aEP. After a 4-h induction of protein expression by 1 mM isopropyl-bD-thiogalactopyranoside (IPTG), the bacterial pellets were collected and detected by 12% sodium dodecyl sulfate-polyacrylamide gel electrophoresis (SDS-PAGE). The recombinant peptide was extracted from inclusion bodies and purified by molecular sieve filtration. Protein concentration was determined by the Coomassie light blue method with bovine serum albumin as the standard.

### Antibody production and purification

The purified aEP of aP2X7R was used as an antigen to immunize mice to produce antiserum [18]. ICR mice (20–22 g) were intraperitoneally immunized with 0.5 ml purified aEP (1 mg ml^−1^) emulsified with an equal volume of Freund’s complete adjuvant. Thereafter, the mice were injected intraperitoneally with the same amount of aEP emulsified with Freund’s incomplete adjuvant on days 14 and 28 post-primary immunization. One day after the final injection, the mice were fasted overnight, and the blood was collected from the caudal vein. After standing at 4°C for 8 h, sera were collected by centrifuging at 14000×g for 10 min at 4°C and stored at −70°C until use. Control mice were injected with the same volume complete Freund’s adjuvant. The specificity of anti-aEP serum was determined by Western blot with macrophage lysates and recombinant aEP protein. Anti-aEP polyclonal antibody (aEPAb) from the generated antisera and control isotype immunoglobulin G (IgG) from control mice were purified by protein G chromatography media (Bio-Rad, Shanghai, China) according to the manufacturer’s protocol. Western blotting was performed to detect the purified recombinant aEP of aP2X7R. The same recombinant protein sample was sequenced to detect the specificity of aEPAb. The protein sample was resolved by SDS-PAGE and subsequently transferred onto a polyvinylidene fluoride (PVDF) membrane. The N-terminal amino acid sequence of the purified aEP of aP2X7R was determined by automated Edman degradation on a PE/ABD Model 470A protein sequencer (Foster City, CA) operated with gas-phase delivery of trifluoroacetic acid.

### Cell culture

Ayu macrophages were isolated as previously described with some modifications [20]. Briefly, head kidneys were aseptically extracted, collected, and meshed in RPMI 1640 (Invitrogen, Shanghai, China) supplemented with 2% fetal bovine serum (FBS) (Invitrogen), penicillin (100 U ml^−1^), streptomycin (100 µg ml^−1^), and heparin (20 U ml^−1^). The cell suspension was centrifuged at 400×g for 20 min on Ficoll (GE Healthcare Life Sciences, New Jersey, USA) at a suspension:Ficoll ratio of 2∶1. Cells were collected from the interphase, washed, and dissolved in RPMI 1640 supplemented with 0.1% FBS and antibiotics. The cells were then seeded into 35-mm well plates at a density of 2×10^6^ cells well^−1^ and allowed to adhere overnight at 24°C in an atmosphere with 5% CO_2_. The medium was changed to complete medium (4% ayu serum, 6% FBS, 100 U ml^−1^ penicillin, 100 µg ml^−1^ streptomycin), and cells were kept in the incubator under the same conditions.

### Infection of macrophages with *L. anguillarum*

Before infection, the medium was changed to antibiotic-free medium, and cells were incubated for another 12 h. Macrophages were infected with live *L. anguillarum* at a multiplicity of infection (MOI) of 10. Infected and uninfected cells were harvested at 0, 4, 8, 12, and 24 hpi. Cell RNA was extracted using RNAiso Reagent (TaKaRa). Simultaneously, cells were also lysed in buffer containing protease inhibitors (20 mM Tris-HCl, 1 mM EDTA, 1% Triton X-100, 1 mM PMSF, 10 mg ml^−1^ aprotinin, 10 mg ml^−1^ leupeptin, and 10 mg ml^−1^ pepstatin-A, pH 8.0), and total proteins were prepared as described previously [21].

### siRNA blocking

aP2X7R siRNA (5′-CGGACAAGGACUGUGUCAAAGGAUU-3′) and a scrambled siRNA (5′-GAGACACAGGCUCGUUAAUAGGAGU-3′) were purchased from Invitrogen. Transfection of cells with siRNA was performed using Lipofectamine 2000 transfection reagent (Invitrogen) according to the manufacture's protocol. Briefly, 5 µl of Lipofectamine 2000 in 250 µl of Opti-MEM (Invitrogen) was mixed with either 100 pmol aP2X7R siRNA or 100 pmol scrambled siRNA in 250 µl of Opti-MEM. The mixture was then incubated for 20 min at room temperature and was added to macrophages with a final siRNA concentration of 40 nM. After a 5.5-h incubation, media were changed to complete media, and cells were cultured for another 48 h before collection for cell death, phagocytosis, and bacterial killing assays. RT-qPCR and Western blotting were used to confirm aP2X7R knockdown.

### Western blot analysis

Protein samples from macrophages subjected to bacterial infection or siRNA blocking were analyzed by SDS–PAGE and Western blotting, as previously reported [18]. aEPAb was used as the primary antibody at 6 µg ml^−1^, and the bound primary antibody was evaluated using the relevant HRP-labeled goat anti-mouse IgG at 160 ng ml^−1^. The proteins were visualized using an enhanced chemiluminescence (ECL) kit (Santa Cruz Biotechnology, Santa Cruz, CA, USA). Changes in relative band intensity were analyzed by the NIH ImageJ program. Three biological replicates were used for each treatment.

### Measurement of cell death

Ayu head kidney-derived macrophages were seeded in 96-well plates (1×10^4^ cell well^−1^) and treated with various concentrations of ATP (0.01, 0.1, 0.5, 1, 1.5, 2.5, 5, and 10 mM) for 30 min at 24°C in an atmosphere with 5% CO_2_. The media were then removed and replaced with complete media. The cells were incubated for an additional 6 h, and cytoplasmic histone-associated DNA fragments were quantified using a Cell Death Detection ELISA^PLUS^ (Roche Applied Science, Indianapolis, USA) according to the manufacturer’s protocol. To assess the effects of aP2X7R on ATP-induced cell death, cells were transfected with aP2X7R siRNA for 48 h, pre-incubated with different concentrations of aEPAb (1, 5, 10, 15, 25, 50, 100, 200, and 500 µg ml^−1^) for 30 min, or pre-treated with various concentrations of oxidized ATP (oATP; 1, 10, 100, 150, 300, 500, and 1000 µM; Sigma, Shanghai, China) for 2 h. oATP is a small Schiff base molecule that has been used as an antagonist of P2X7R [22]. Scrambled siRNA, mouse isotype IgG, and PBS were added as controls. Cells were treated with 5 mM ATP for 30 min, followed by a 6-h incubation in the absence of ATP. Cell death was determined as described above.

### Phagocytosis assay

DH5α *E. coli* in the logarithmic phase of growth were labeled with fluorescein isothiocyanate (FITC; cells were hereafter designated as FITC-DH5α; Sigma) according to the manufacturer’s protocol. Ayu macrophages grown on cover slips in 6-well plates (2×10^6^ cell well^−1^) were transfected with aP2X7R siRNA for 48 h, pre-incubated with 200 µg ml^−1^ aEPAb for 30 min or 300 µM oATP for 2 h. As a control, scrambled siRNA, mouse isotype IgG, and PBS were added. FITC-DH5α were added at an MOI of 10, and cells were further incubated for 30 min. Then, cells were washed extensively with sterile PBS to remove extracellular particles. Trypan blue (0.4%) was used to quench the fluorescence that resulted from particles, which were outside of the cells or sticking to the surface of the cells. The engulfed bacteria were examined by fluorescence microscopy (600× magnification; Nikon Eclipse Ti-U, Tokyo, Japan). The mean fluorescence intensity (MFI) of bacteria engulfed by cells among siRNA, aEPAb, and oATP treatments was analyzed by the NIH ImageJ program, and at least 400 macrophages were counted for each independent assay. The results were expressed as the percent MFI of the control and were shown as the mean ± SEM of a typical example from at least three independent experiments.

### Bacterial killing assay

Ayu macrophages were transfected with aP2X7R siRNA for 48 h, pre-incubated with 200 µg ml^−1^ aEPAb for 30 min or 300 µM oATP for 2 h, and then infected with live *L. anguillarum* at an MOI of 10 as described above. As a control, scrambled siRNA, mouse isotype IgG, and PBS were added. Bacterial phagocytosis was allowed to proceed for 30 min at 24°C in an atmosphere of 5% CO_2_, and the noninternalized *L. anguillarum* were removed by washing with sterile PBS. One set of samples (the uptake group) was lysed in 1% Triton X-100 solution and plated onto solid thiosulfate citrate bile salts sucrose (TCBS) agar medium to provide bacterial uptake values. The remaining set (the kill group) was incubated with 5 mM ATP (Sigma) for a 30 min pulse. The ATP was then removed and replaced with an equal volume of fresh medium, and the cells were further incubated for 1.5 h to allow bacterial killing to occur. Cell lysate-bacterial samples were collected and pelletted at 14000 rpm for 20 min, lysed in 1% Triton X-100 solution, and then plated onto TCBS agar medium. After incubation at 30°C for 18 h, we counted the CFU in the plates. After aEPAb or isotype IgG incubation, cells were treated with a 30-min ATP plus, then cultured in the absence of ATP and harvested at different time intervals (0.5, 1, 2, and 4 h) to investigate whether the bacteria were killed or could not replicate [23]. Bacterial survival was determined by dividing the CFU in the kill group by the CFU in the uptake group. Three independent experiments were performed.

## Results

### AP2X7R gene analysis

**Figure 1:**
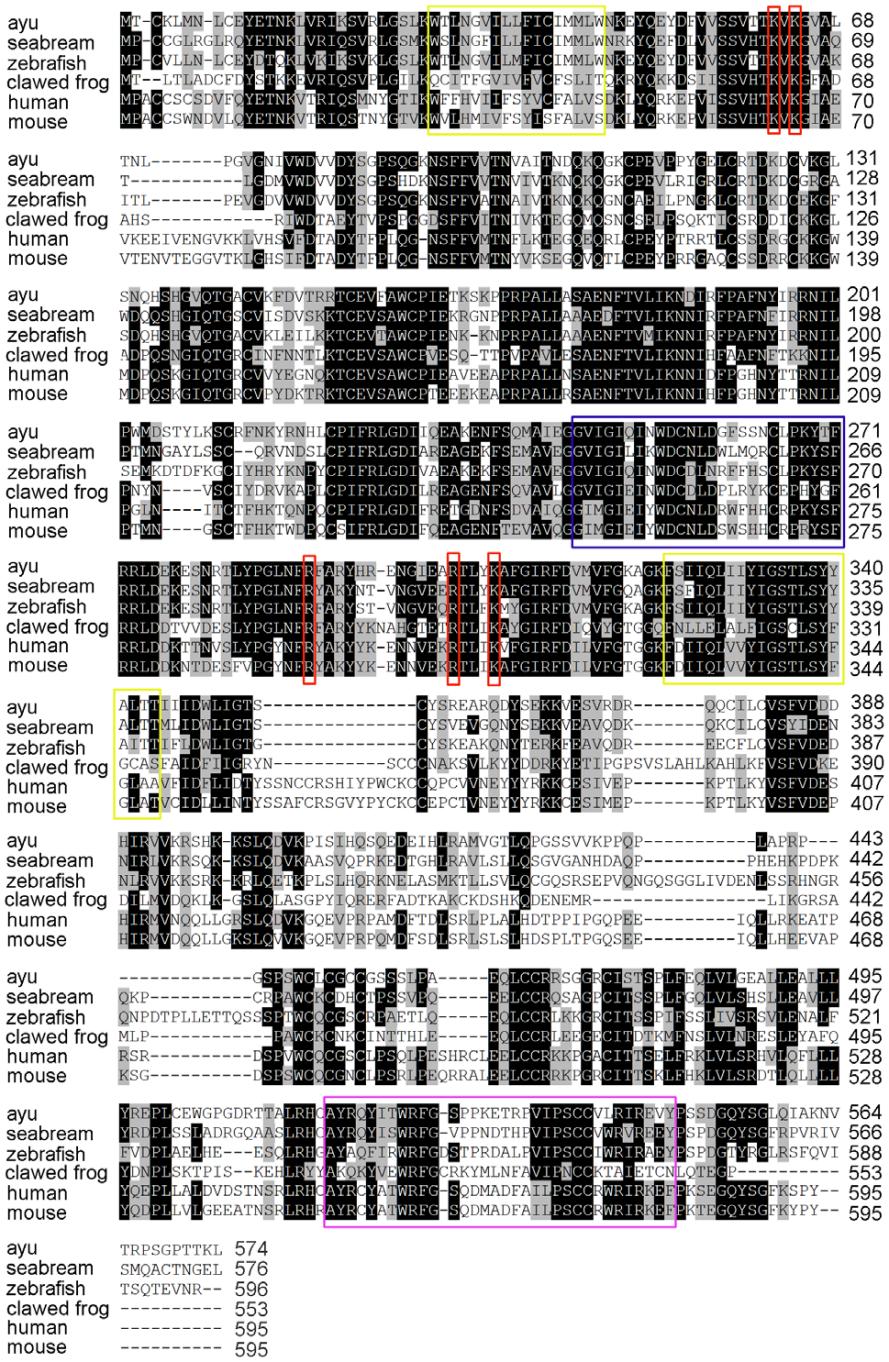
Multiple alignment of vertebrate P2X7R. Threshold for shading was > 60% of similarity. Similar residues are shadowed gray and identical residues are shadowed black. Two predicted transmembrane domains (yellow), the P2X family signature (blue), five residues important for nucleotide binding (red), and the LPS/lipid-binding domain (pink) are shown. The accession numbers of P2X7R sequences are HE984576 for ayu (*P. altivelis*), AJ887997 for gilthead seabream (*S. aurata*), AY292647 for zebrafish (*D. rerio*), AJ345114 for clawed frog (*X. laevis*), Q99572 for human (*H. sapiens*), and AJ489297 for mouse (*M. musculus*).

**Figure 2:**
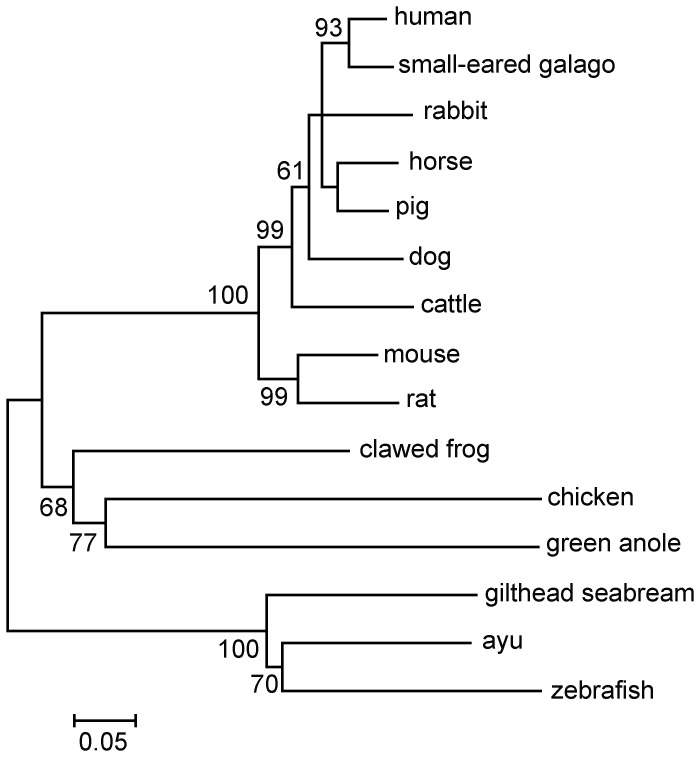
Phylogenetic analysis of aP2X7R amino acid sequences using the neighbor-joining method. The values at the forks indicate the percentage of trees in which this grouping occurred after bootstrapping (1000 replicates). The scale bar shows the number of substitutions per base. The accession numbers of sequences used are human (*H. sapiens*), Q99572; small-eared galago (*O. garnettii*) XM_003795998; dog (*C. familiaris*), NM_001113456; horse (*E. caballus*), XM_001495572; pig (*S. scrofa*), XM_001926804; cattle (*B. Taurus*), NM_001206516; rabbit (*O. cuniculus*), XM_002719745; mouse (*M. musculus*), AJ489297; rat (*R. norvegicus*), NM_019256; clawed frog (*X. laevis*), AJ345114; chicken (*G. gallus*), XM_001235162; green anole (*A. carolinensis*), XM_003222779; gilthead seabream (*S. aurata*), AJ887997; ayu (*P. altivelis*), HE984576; and zebrafish (*D. rerio*), AY292647.

The full-length cDNA of aP2X7R, measuring 2046 nucleotides (nts) long, was obtained and deposited into GenBank with the accession number HE984576. The 1725-nt open reading frame of aP2X7R encoded a polypeptide of 574 amino acids corresponding to a calculated molecular weight (MW) of 65.0 kDa. The deduced protein contained no putative signal peptide predicted by the SignalP 4.0 program, suggesting that it was not a secretory protein. Multiple alignment revealed that a high level of conservation of the P2X receptor family signature, the two transmembrane domains and a long C-terminal domain, were present in aP2X7R (Fig. 1). Five residues important for nucleotide binding in mammalian P2X7R molecules [24], [25] were intact in aP2X7R (Fig. 1). Sequence analysis showed that aP2X7R had the highest amino acid identity to P2X7R from the gilthead seabream (67%). Phylogenetic tree analysis showed that P2X7R proteins from the ayu and other fish were grouped together, forming a fish cluster distinct from the mammalian cluster (Fig. 2).

### Preparation and purification of an antibody against aEP

**Figure 3:**
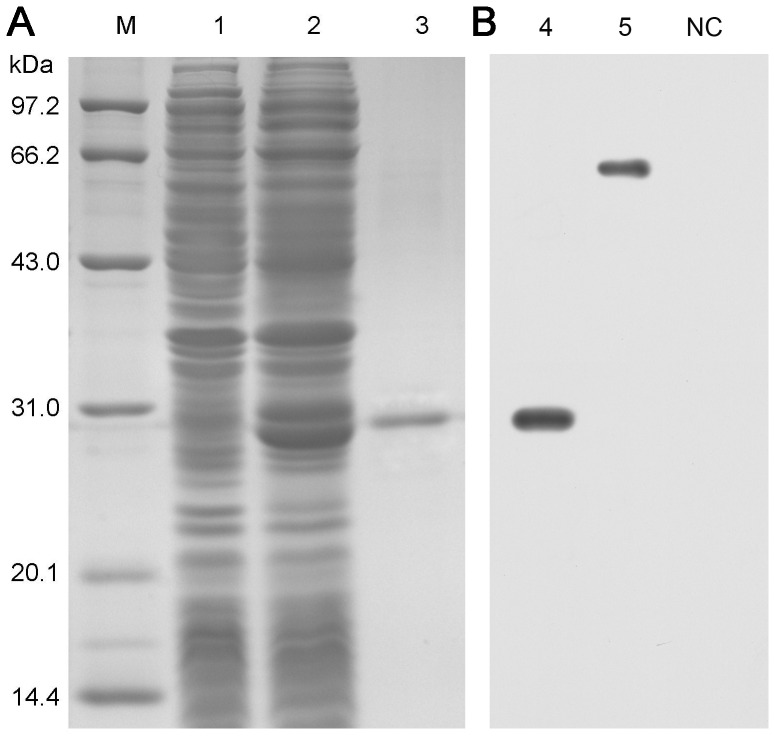
Bacterial expression of aEP and preparation of antiserum. (A) SDS-PAGE analysis of recombinant aEP. Lane M: protein marker; 1 and 2: protein from BL21 *E. coli* transformed with the pSBET-aEP plasmid before and after IPTG induction; 3: purified recombinant protein. (B) Western blot analysis of recombinant aEP and native aP2X7R. Lane 4: purified recombinant protein; 5: ayu head kidney-derived macrophages; NC: negative control, BL21 lysate before IPTG induction.

aEP (comprising amino acids 60–310 of the aP2X7R ectodomain peptide) was overexpressed in BL21 (DE3) *E. coli* transformed with the pSBET-aEP plasmid (Fig. 3A, lane 2). Protein from BL21 (DE3) *E. coli* transformed with the pSBET-aEP plasmid before and after IPTG induction was shown in Fig. 3A (lanes 1 and 2, respectively). The recombinant peptide was extracted from inclusion bodies and purified by molecular sieve filtration. As shown by SDS-PAGE analysis, the recombinant aEP was highly pure (Fig. 3A, lane 3). Using our anti-aEP polyclonal antibody, which was generated by immunizing mice, we detected aEP by Western blotting. The MW of recombinant aEP was 30.0 kDa, while that of the full-length aP2X7R protein from macrophages was 65.0 kDa (Fig. 3B). The specificity of the antibody was verified by sequencing the band from the recombinant protein detected by Western blotting. The partial N-terminal amino acid sequence was identified as MTKVKGVALTNLPGVGNIVW, which was indeed the N-terminal sequence of the aEP of aP2X7R. Antibody was subsequently purified by protein G chromatography media and stored at −70°C before the following study.

### Alteration of tissue and macrophage aP2X7R mRNA expression upon *L. anguillarum* infection

**Figure 4:**
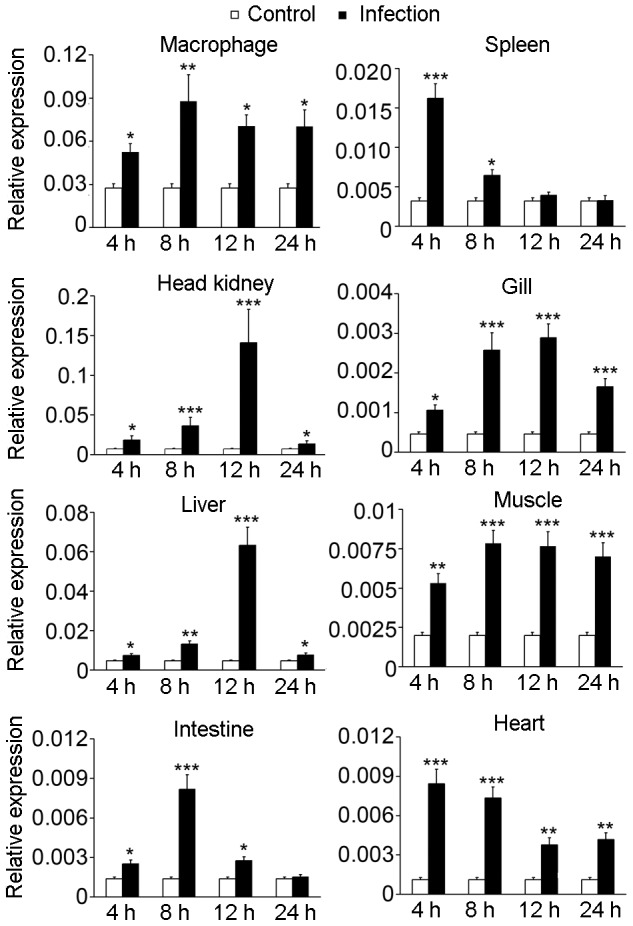
RT-qPCR analysis of aP2X7R mRNA expression following bacterial infection in various tissues and macrophages. Fish or macrophages were infected with *L. anguillarum* for 4, 8, 12, or 24 h. aP2X7R transcript levels were normalized by the β-actin content at the same time point. Each bar represents the mean ± SEM of the results from four independent experiments. *P<0.05; **P<0.01; ***P<0.001.

aP2X7R transcripts were detected in macrophages and tissues, including the spleen, head kidney, gill, liver, muscle, intestine and heart, by RT-qPCR (Fig. 4). The expression levels of the receptor were higher in macrophages, as well as in the head kidney, liver, and spleen, as compared to the other studied tissues. After challenge with *L. anguillarum*, the aP2X7R transcripts in these tissues showed a time-dependent increase in expression pattern. The bacterial infection increased the expression of aP2X7R mRNA in all examined tissues at 4 hpi. The most dramatic upregulation in aP2X7R mRNA expression was observed in the head kidneys (up to 19.99-fold) at 12 hpi (Fig. 4).

### Expression of macrophage aP2X7R upon *L. anguillarum* infection

**Figure 5:**
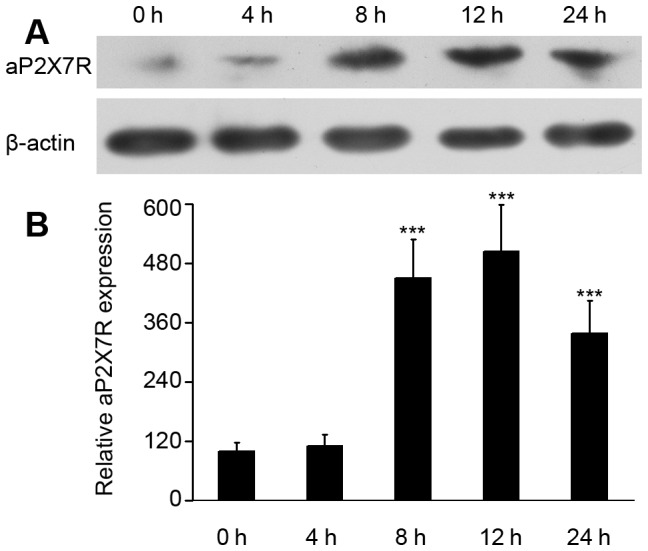
Western blot analysis of aP2X7R protein expression in macrophages upon *L. anguillarum* treatment. (A) Protein collected from *L. anguillarum*-infected macrophages at 0, 4, 8, 12 and 24 hpi was assayed by Western blotting using an antiserum specific to aP2X7R. (B) Histogram displaying the changes in relative band intensity of aP2X7R protein in *L. anguillarum*-infected macrophages at 0, 4, 8, 12, and 24 h. Data are representative of three independent experiments. ***P<0.001.

To further analyze the protein levels of aP2X7R in ayu macrophages upon *L. anguillarum* infection, protein was analyzed by Western blotting using specific aEPAb. After *L. anguillarum* challenge, aP2X7R protein was increased at 8 hpi, peaked (5.05-fold increase) at 12 hpi, and remained at a significantly higher level at 24 hpi (3.38-fold increase), as compared to that at 0 hpi (Fig. 5).

### AP2X7R regulated ATP-induced macrophage cell death

**Figure 6:**
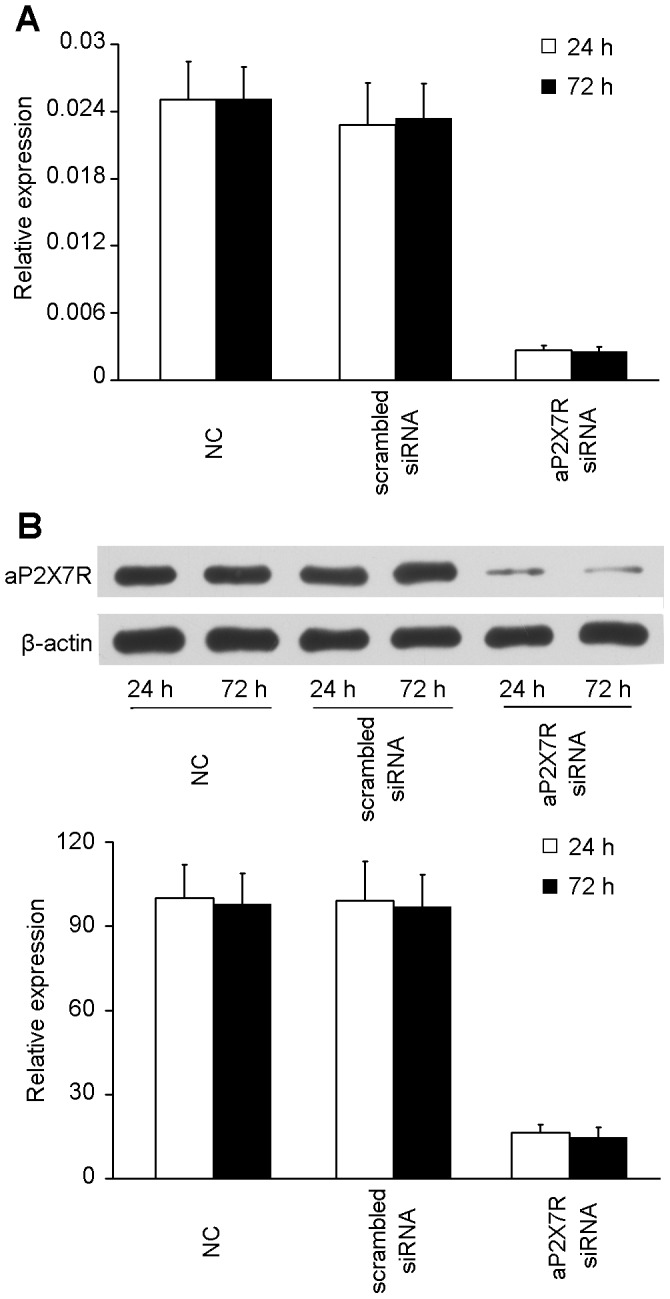
RT-qPCR and Western blot analysis of aP2X7R expression following siRNA transfection. aP2X7R siRNA was transfected into macrophages for 48 or 72 h. Scrambled siRNA was transfected as a control. (A) Histogram displaying the *aP2X7R* mRNA expression following siRNA transfection by RT-qPCR analysis. (B) The effects of aP2X7R siRNA transfection on knockdown of aP2X7R protein was confirmed by Western blot analysis. Histogram displays the changes in relative band intensity of aP2X7R protein upon siRNA treatment. Data are representative of three independent experiments. NC: negative control without any siRNA.

**Figure 7:**
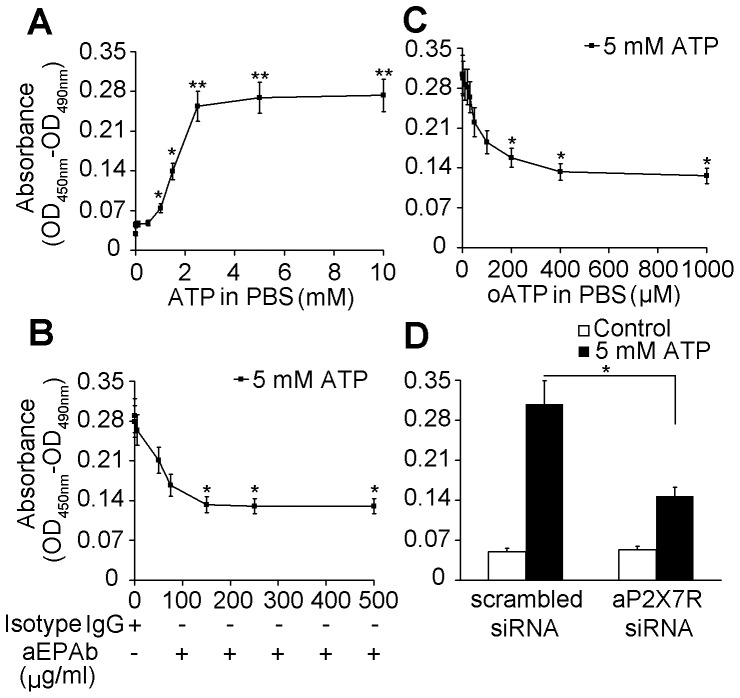
ATP induced cell death through activation of aP2X7R. (A) Cell death induced by different concentrations of ATP. Macrophages were incubated with PBS (control) or various concentrations of ATP for 30 min and cultured for an additional 6 h without ATP. (B) The effects of aEPAb on ATP-induced cell death. Cells were pre-incubated with different concentrations of aEPAb for 30 min before ATP treatment. (C) The effects of oATP on ATP-induced cell death. The cells were pretreated with various concentrations of oATP for 2 h before ATP treatment. (D) The effects of aP2X7R siRNA on ATP-induced cell death. The cells were transfected with siRNA for 48 h before ATP treatment. Mouse isotype IgG, PBS, and scrambled siRNA were added as controls. Cells were treated with ATP for 30 min, followed by a 6-h incubation in the absence of ATP. Data are representative of three independent experiments. *P<0.05; **P<0.01.

An RNAi assay was performed to knockdown aP2X7R expression. When the cells were transfected with aP2X7R siRNA, the mRNA and protein levels of aP2X7R were significantly downregulated as compared to corresponding mRNA and protein levels in cells transfected with scrambled siRNA and negative control cells (Fig. 6A and B). We measured cell death in terms of cytoplasmic histone-associated DNA fragment formation. Treatment with ATP induced cell death in a dose-dependent manner (Fig. 7A); ATP concentrations below 1 mM had little effect, and the ATP concentration evoking half-maximal cell death effect (EC50) was 1.5 mM (Fig. 7A). The blocking activity of aEPAb and oATP on ATP-induced cell death also showed a dose-dependent effect (Fig. 7B and C). Knockdown of aP2X7R by siRNA inhibited ATP-induced cell death (Fig. 7D). These results confirmed that ATP-induced cell death was mediated by aP2X7R.

### AP2X7R mediated the phagocytosis and bacterial killing of ayu macrophages

**Figure 8:**
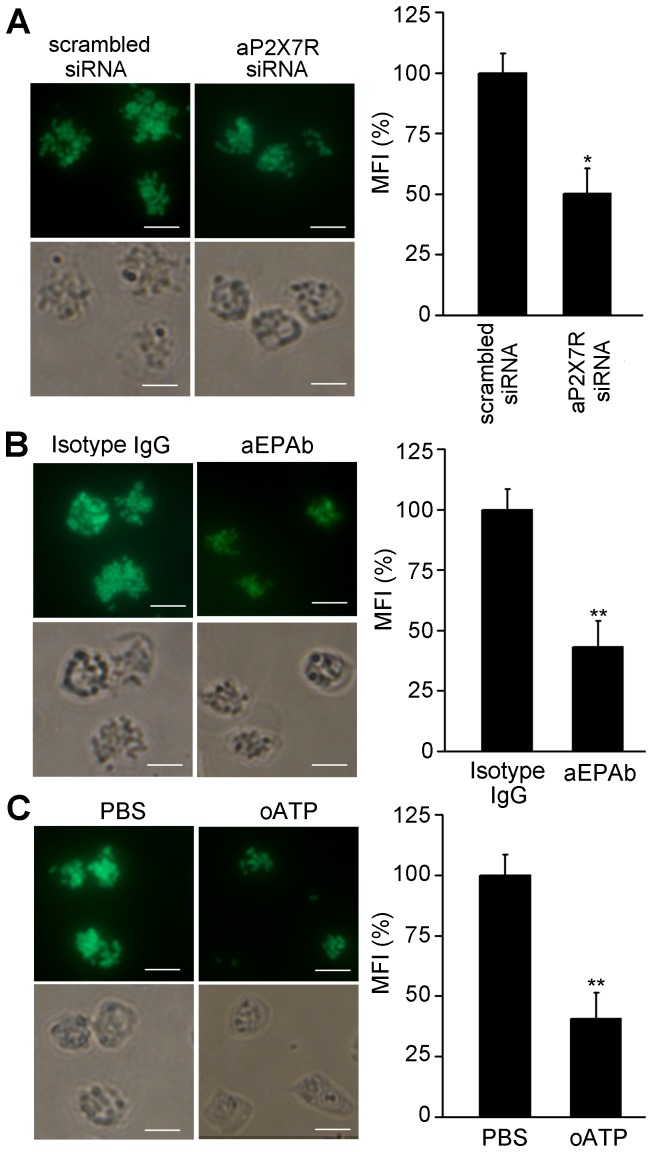
The phagocytosis of ayu macrophages after aP2X7R ablation. Fluorescence images of phagocytosis of FITC-DH5α in macrophages treated with siRNA (A), aEPAb (B), and oATP (C). After incubation with siRNA for 48 h, aEPAb for 30 min, or oATP for 2 h, macrophages were incubated with FITC-DH5α at an MOI of 10 for an additional 30 min. Scrambled siRNA, mouse isotype IgG, and PBS were added as controls. Histogram represents the percent mean fluorescence intensity (MFI) of bacteria engulfed by cells treated with siRNA, aEPAb, or oATP. Data are representative of at least three independent experiments. Scale bar, 10 µm. *P<0.05; **P<0.01.

**Figure 9:**
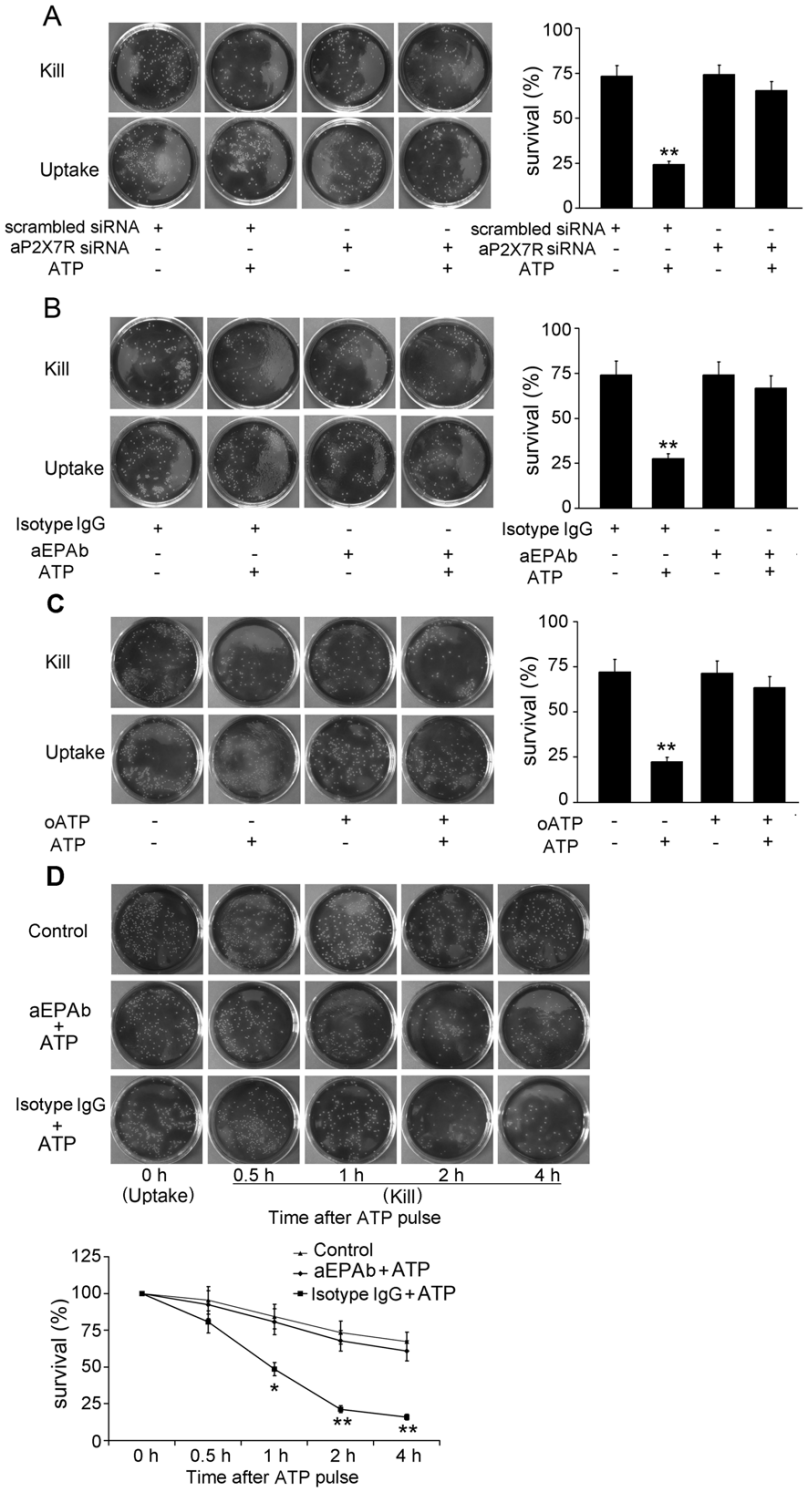
ATP-induced bacterial killing activity was inhibited after aP2X7R ablation. Plates displayed the survival *L. anguillarum* from macrophages treated with siRNA (A), aEPAb (B), or oATP (C). The histogram demonstrated the effects of siRNA, aEPAb, or oATP on bacterial killing. Macrophages were infected with live *L. anguillarum* after siRNA, aEPAb, or oATP treatment. *L. anguillarum* viability was examined through CFU assay after ATP treatment. Mouse IgG, scrambled siRNA, and PBS were added as controls for the respective treatments. (D) Reduction of *L. anguillarum* viability induced by ATP. After treatment with aEPAb or isotype IgG, cells were treated with ATP. Subsequently, cells were harvested at different time intervals, and bacterial viability was monitored through CFU assays. Data are representative of at least three independent experiments. *P<0.05; **P<0.01.

The phagocytosis of FITC-DH5α was significantly downregulated to approximately 50.33% of cells transfected with the scrambled siRNA control (Fig. 8A). Moreover, aP2X7R blockage by aEPAb and oATP also altered the phagocytosis of ayu macrophages (Fig. 8B and C). Next, bacteria survival was determined by the CFU counting method to assess the bacterial killing of ayu macrophages (Fig. 9). aP2X7R siRNA transfection significantly inhibited ATP-induced bacterial killing in ayu macrophages (Fig. 9A). Blockage with aEPAb and oATP also suppressed the effects of ATP on the bacterial killing of ayu macrophages (Fig. 9B and C). Kinetic evaluation of bacterial killing by ATP-pulsed macrophages was performed. At 1 h after ATP stimulation, there was a significant reduction in bacterial viability of the control, further decreasing at 2 and 4 h after ATP stimulation, which could be inhibited by aEPAb (Fig. 9D). These results further confirmed that aP2X7R could mediate phagocytosis and bacterial killing of ayu macrophages.

## Discussion

In this study, we provided the full-length sequence of the aP2X7R gene. The deduced protein possessed several common features shared by other P2X7R homologues [1]. Sequence comparison and phylogenetic tree analysis also confirmed aP2X7R as a distinct member of the fish purinergic subtype receptor. These data indicated that aP2X7R is homologue to mammalian P2X7R and may play a role in ayu innate immunity. Multiple alignment of fish, amphibian (clawed frog), and mammalian P2X7R sequences revealed a high level of conservation of the P2X7R family signature, the two transmembrane domains and the long C-terminal domain. Five residues important for nucleotide binding in mammalian P2X7R were also present in a conserved position in the fish and amphibian proteins. The conserved structures of P2X7R in evolution indicated that P2X7R may play important roles in both fish and mammals.

P2X7R has a ubiquitous tissue distribution, and its expression levels may vary over orders of magnitude in mammals [2]. It is predominantly expressed in immune cells from myeloid lineages, such as macrophages, monocytes, and dendritic cells [3], [26]. The head kidney, thymus, spleen, and mucosa-associated lymphoid tissues are known to be the major lymphoid tissues in teleost fish [27]. In this study, aP2X7R transcripts were widely distributed in ayu tissues and were especially abundant in the head kidney, spleen, and liver. Similarly, aP2X7R mRNA was abundant in ayu head kidney-derived macrophages. These results suggest that aP2X7R is also mainly expressed in immune organs and cells. Mammalian P2X7R has been found upregulated after infection [9], [21], [28]. A significant increase in the expression of P2X7R has been observed on human macrophages infected with *M. tuberculosis* [9] or mouse macrophages infected with *Leishmania amazonensis* [21]. A significant release of ATP has also been detected by *M. tuberculosis*-infected macrophages [9]. In our study, aP2X7R was upregulated in all examined ayu tissues after bacterial infection, suggesting that aP2X7R is implicated in ayu infection response.

In mammals, it has been demonstrated that prolonged stimulation of macrophages using high concentrations of ATP causes cell death via P2X7R-induced apoptosis [9], [29]. Cell death by ATP stimulation proceeds through apoptotic nuclear alterations, such as chromatin condensation and DNA fragmentation, but not cytolytic or membrane damage and occurs concomitant with a decrease in bacterial viability [8], [9], [30]. However, it is still unclear whether ATP activates P2X7R to induce cell death in fish. In the current study, ayu macrophage death was observed following treatment with ATP. The EC_50_ concentration of ATP needed to induce aP2X7R-dependent cell death was 1.5 mM, which seemed higher than the concentration of ATP required in their mammalian counterparts [29]. We also found that ATP-induced cell death was suppressed after aP2X7R blockage via RNAi, aEPAb, and oATP. Our data suggest ATP activates aP2X7R to induce the macrophage cell death in ayu, a teleost.

P2X7R has also been reported to be involved in phagocytosis and clearance of bacteria by macrophages [10], [31]. Phagocytosis is inhibited after ATP dissociates myosin IIA from P2X7R complex [10], [32], [33]. However, in the absence of ATP, P2X7R may function in phagocytosis [34]. Furthermore, the mechanisms underlying P2X7R and phagocytosis in the absence of ATP have also been defined [35]. A peptide mimicking the extracellular domain of P2X7R can bind phagocytosed particles, suggesting that P2X7R mediates phagocytosis via direct recognition of the particles [35]. In the present study, we observed that siRNA, specially designed to knockdown the expression of aP2X7R, aEPAb (the anti-aP2X7R extracellular domain antibody), and oATP (a P2X7R antagonist), could significantly attenuate the phagocytic activity of ayu macrophages. Therefore, aP2X7R may mediate phagocytosis as a scavenger receptor in ayu macrophages.

After infection, intracellular bacterial viability is reduced after ATP is released into the extracellular media [9]. Here, we found that the survival of bacteria was downregulated in ATP induced macrophages compared with negative control. Moreover, the number of CFUs was confirmed to be reduced after the cells were treated with ATP for different times. This result suggests that bacteria may actually be killed in macrophages treated with ATP rather than just exhibit halted replication. As shown in multiple studies, P2X7R mediates this process of ATP-induced bacterial killing of macrophages [8], [9], [36]_ENREF_30. Many studies have also suggested that killing of bacteria and other pathogens via the P2X7R-mediate pathway is independent of nitric oxide (NO) [8], [37]. The death of host cells may explain this method of bacterial killing [8], [21], [38], [39]. We also found that ATP induced cell death in ayu macrophages, suggesting that ATP-induced bacterial killing may also result from cell death. Furthermore, ATP-induced bacterial killing was inhibited by aP2X7R knockdown by siRNA or blockage by aEPAb and oATP. Thus, we speculate that aP2X7R may be involved in bacterial killing in ayu macrophages.

In conclusion, we found that aP2X7R was mainly distributed in immune tissues of the ayu. Moreover, upon bacterial challenge, aP2X7R mRNA was significantly upregulated in all tested tissues. The mRNA and protein levels of aP2X7R were also significantly increased in macrophages in response to bacterial infection. In the seabream, a teleost fish, activation of P2X7R regulates phosphatidylserine externalization and cell permeabilization, but fails to induce IL-1β release in leukocytes [14]. However, the function of P2X7R in other fish species remains poorly understood. Our data further demonstrate that aP2X7R may regulate cell death, phagocytosis, and bacterial killing in ayu macrophages in response to bacterial infection, suggesting a conserved function for P2X7R in macrophage modulation.
